# INPP5E Regulates the Distribution of Phospholipids on Cilia in RPE1 Cells

**DOI:** 10.1002/jcla.25031

**Published:** 2024-03-21

**Authors:** Denghui Zhai, Lamei Li, Cheng Chen, Xue Wang, Ruming Liu, Ying Shan

**Affiliations:** ^1^ State Key Laboratory of Medicinal Chemical Biology, College of Life Sciences Nankai University Tianjin China

**Keywords:** ciliary base, INPP5E, PI(4,5)P_2_, PI4P, primary cilia

## Abstract

**Background:**

Primary cilia are static microtubule‐based structures protruding from the cell surface and present on most vertebrate cells. The appropriate localization of phospholipids is essential for cilia formation and stability. INPP5E is a cilia‐localized inositol 5‐phosphatase; its deletion alters the phosphoinositide composition in the ciliary membrane, disrupting ciliary function.

**Methods:**

The EGFP‐2xP4M^SidM^, PH^PLCδ1^‐EGFP, and SMO‐tRFP plasmids were constructed by the Gateway system to establish a stable RPE1 cell line. The *INPP5E* KO RPE1 cell line was constructed with the CRISPR/Cas9 system. The localization of INPP5E and the distribution of PI(4,5)P_2_ and PI4P were examined by immunofluorescence microscopy. The fluorescence intensity co‐localized with cilia was quantified by ImageJ.

**Results:**

In RPE1 cells, PI4P is localized at the ciliary membrane, whereas PI(4,5)P_2_ is localized at the base of cilia. Knocking down or knocking out *INPP5E* alters this distribution, resulting in the distribution of PI(4,5)P_2_ along the ciliary membrane and the disappearance of PI4P from the cilia. Meanwhile, PI(4,5)P_2_ is located in the ciliary membrane labeled by SMO‐tRFP.

**Conclusions:**

INPP5E regulates the distribution of phosphoinositide on cilia. PI(4,5)P_2_ localizes at the ciliary membrane labeled with SMO‐tRFP, indicating that ciliary pocket membrane contains PI(4,5)P_2_, and phosphoinositide composition in early membrane structures may differ from that in mature ciliary membrane.

## Graphical abstract

INPP5E regulates the distribution of phosphoinositide (PI) on cilia. PI(4,5)P_2_ is localized at the ciliary membrane labeled with SMO‐tRFP. This suggests that the PI composition in early membrane structures may differ from that in mature ciliary membrane.

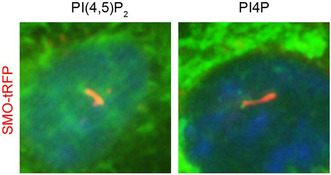

## 1 Introduction

The primary cilium is a microtubule‐based specialized organelle protruding from the cell membrane surface. Operating as a cellular antenna, it can perceive changes in external mechanical and chemical signals, triggering cellular responses and contributing to embryogenesis, sensory perception, and tissue homeostasis [1, 2, 3, 4]. Primary cilia consist of four essential components: basal body, transition zone, axoneme, and ciliary membrane, with microtubules being the core of cilia and playing a decisive role in stability [5, 6, 7, 8, 9]. Structural and functional abnormalities in primary cilia lead to various diseases, such as polydactyly, infertility, obesity, retinal degeneration, and polycystic kidney disease, collectively known as “ciliopathies.” [10, 11, 12] The latest research shows that primary cilia are closely related to liver fibrosis, and the loss of cilia will aggravate the process of liver fibrosis [13]. Additionally, blocking HDAC6‐mediated cilium disassembly protects mice from retinopathy of prematurity (ROP)‐associated retinal defects [14].

Phosphoinositide (PI), a lipid molecule constituting only 10%–15% of membrane phospholipids, plays a pivotal role in cell surface signal transduction, membrane transport, and the regulation of cytoskeletal dynamics [15, 16, 17]. While the ciliary membrane is structurally continuous with the plasma membrane, it exhibits a unique PI composition. The PI on the ciliary membrane is predominantly PI(4)P, while PI(4,5)P_2_ is localized to the transition zone [18, 19]. This PI distribution is mediated by INPP5E. INPP5E is an inositol polyphosphate 5‐phosphatase that is mainly located in cilia of quiescent cells to maintain its function and stability [20, 21, 22, 23]. In addition, INPP5E is also located in lysosomes, and its membrane anchoring and enzymatic activity are required for the autophagy process [24, 25, 26]. Depletion of INPP5E disrupts Hedgehog signaling and transition zone function by altering PI distribution in cilia [18, 19, 27]. Elevated PI(4,5)P_2_ levels caused by INPP5E depletion contribute to actin polymerization and the secretion of ciliary ectosomes. This process facilitates the cilia disassembly and cell cycle reentry and is essential for cilium homeostasis [28, 29].

However, although current research has unveiled the distribution of PI on the mature ciliary membrane, the PI composition of early‐stage ciliary membrane structures remains unreported. Therefore, we established an RPE1 cell line with specific recognition of PI4P and PI(4,5)P_2_ to investigate the PI composition of membrane structures during the early stages of ciliogenesis.

## 2 Materials and Methods

### 2.1 Plasmids and siRNA

Mammalian expression plasmid for SMO‐tRFP was constructed by the insertion of the respective cDNAs into the pDONR vector and then recombined into ptRFP destination vectors. Details of other constructs were described previously, including EGFP‐2xP4M^SidM^ (GFP‐conjugated P4M domain consisting of residues 546–647 of *Legionella pneumophila* SidM [30]) and PH^PLCδ1^‐EGFP (GFP‐tagged PH domain and phospholipase C δ, which is known to specifically bind PI(4,5)P_2_ [31]). The siRNAs against human INPP5E (siINPP5E) with a sequence of 5′‐GGAAUUAAAAGACGGAUUU‐3′ [32] and the negative control siRNAs (siControl) were synthesized by QingKe.

### 2.2 Cell Culture and Transfection

hTERT‐RPE1 cells were cultured in DMEM/F12 (Gibco, ThermoFisher), and HEK293T cells were cultured in DMEM (Gibco, ThermoFisher) supplemented with 10% fetal bovine serum (ExCell Bio), 100 units/mL penicillin, and 100 mg/mL streptomycin. All cell types were maintained at 37°C in a 5% CO_2_ atmosphere. Plasmids and siRNA were transfected into RPE1 or HEK293T cells using Lipofectamine 3000 (Invitrogen) or Lipofectamine RNAiMAX (Life) according to the manufacturer's protocol. For cilia induction, approximately 70% of the cells at confluence were altered to the corresponding medium without serum to induce cilia formation.

### 2.3 Construction of Stable Expression Cell Line

Expression plasmids were co‐transfected with the viral packaging plasmid psPAX2 and the envelope plasmid pMD2.G into 293T cells. The viral supernatant was subsequently filtered through 0.22‐μm filters and applied (200 μL) to 6‐well plates. Cells were cultured in a selection medium containing puromycin (3 μg/mL) or blasticidin (10 μg/mL) when incubated for 48 h. The infected cells were selected for 2 weeks and confirmed by immunoblots or immunofluorescence. Different tag‐positive cells were sorted to establish the final fluorescent cell lines.

### 2.4 CRISPR/Cas9 Knockout Cell Lines

The CRISPR/Cas9 system was used to generate RPE1 INPP5E knockout cell lines as previously described [33]. The gRNA targeting the sequence of 5′‐GAGCAGCACCCATCGCCCCG‐3′ in INPP5E was designed based on on‐target and off‐target‐scores using the webtool Benchling (https://benchling.com) and inserted into the plasmid Lenti‐CRISPR‐V2, which also contains expression cassettes for Cas9 and a puromycin selectable marker. After correct sequencing, it co‐transfected with viral packaging plasmid psPAX2 and envelop plasmid pMD2.G into 293T cells, and viral fluids were collected to infect RPE1 cells and screened for effective infection.

### 2.5 Immunofluorescence

Cells were seeded on slides in 24‐well plates and cultured without serum for 48 h to induce cilia formation. Immunofluorescence of cells was performed by fixing in 4% paraformaldehyde for 15 min and then permeating with 0.1% Triton X‐100 (Sigma‐Aldrich) for 15 min. Then, 1% BSA (Sigma‐Aldrich) was used to block at room temperature for 15 min. Subsequently, slides were incubated with primary antibodies for 1 h at room temperature or overnight at 4°C. The following primary antibodies were used: mouse anti‐acetylated α‐tubulin (Sigma‐Aldrich, T7451) and rabbit anti‐ARL13B (Proteintech, 17711‐1‐AP), and then, slides were incubated with secondary antibodies and DAPI at room temperature. Alexa Fluor 488‐ or 568‐conjugated secondary antibodies were purchased from Life Technologies. Cells were examined with a Zeiss LSM710 confocal microscope. The fluorescent intensity was quantified by ImageJ.

### 2.6 Quantification and Statistical Analysis

Quantitative statistics and mapping were completed using the ImageJ software and the GraphPad Prism software.

## 3 Results

### 3.1 PI4P and PI(4,5)P_2_ Respectively Locating at the Cilia and Ciliary Base

**FIGURE 1:**
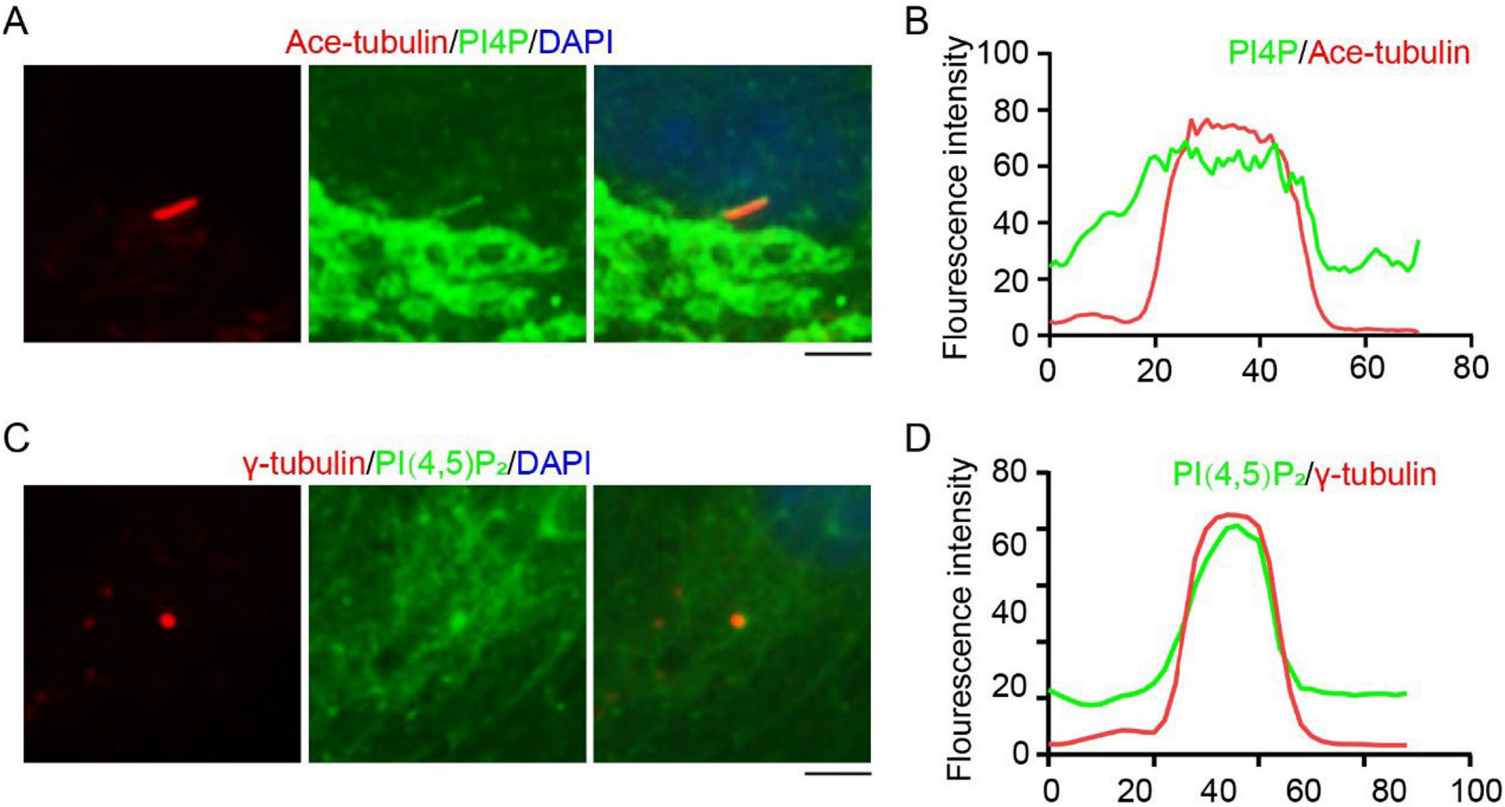
Localization of PI4P and PI(4,5)P_2_ on cilia in RPE1 cells. (A) Immunofluorescence detected the localization of PI4P in RPE1 cells cultured in serum‐free medium for 48 h. (B) The fluorescence intensity of PI4P and Ace‐tubulin in Panel A was assessed using ImageJ. (C) Immunofluorescence detected the localization of PI(4,5)P_2_ in RPE1 cells cultured in serum‐free medium for 48 h. (D) The fluorescence intensity of PI(4,5)P_2_ and γ‐tubulin in Panel B was assessed using ImageJ. Scale bar, 10 μm.

We first attempted to determine the location of PI4P and PI(4,5)P_2_ on cilia and generated RPE1 cells with stable expression‐specific PI‐binding domains fused with EGFP. We found that PI4P co‐localized with cilia (Figure 1A,B), while PI(4,5)P_2_ localized at ciliary base (Figure 1C,D). This suggests that PI4P and PI(4,5)P_2_ are distributed in different locations on cilia.

### 3.2 Validation of the Localization of INPP5E on the Primary Cilia of RPE1 Cells

**FIGURE 2:**
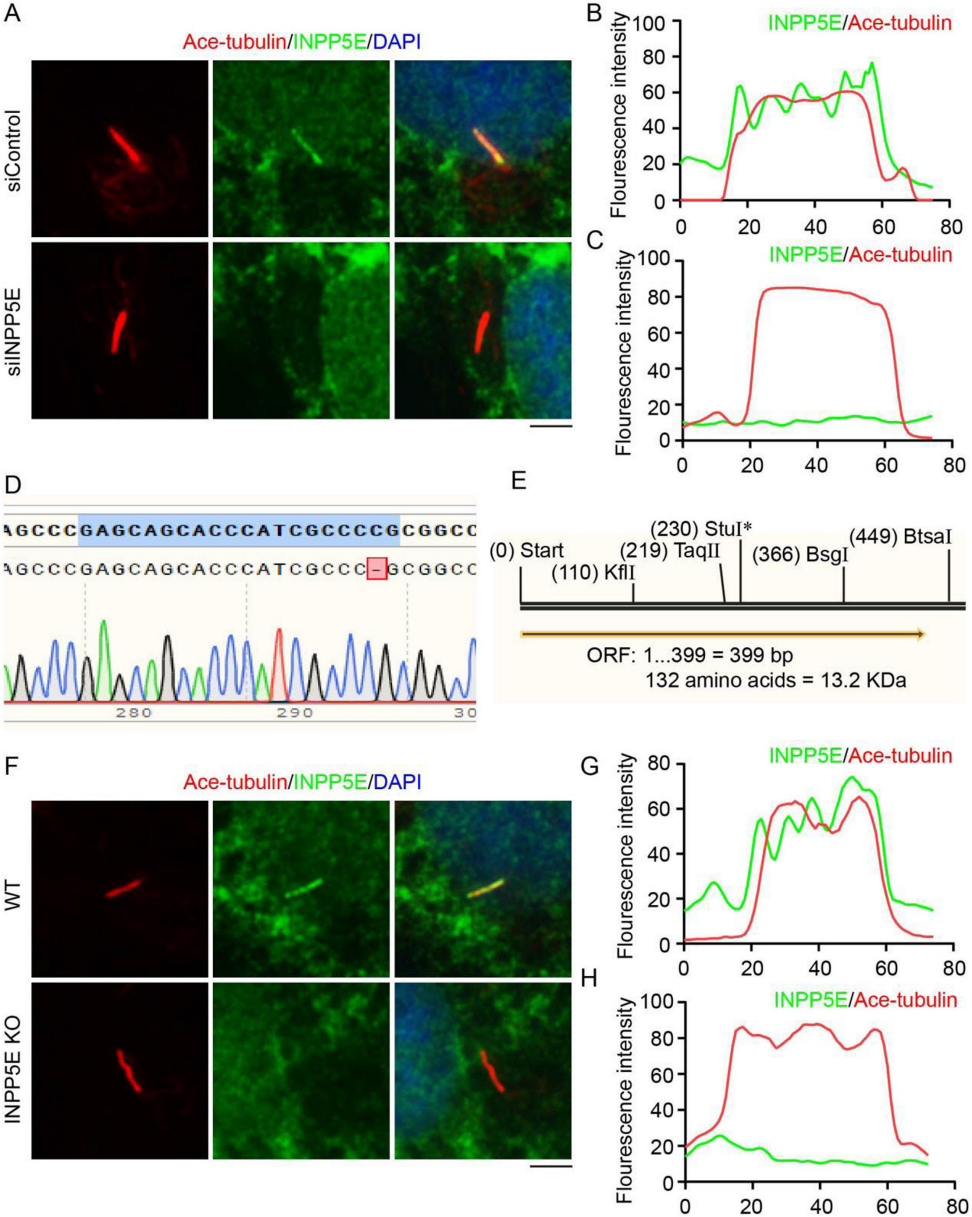
Localization of INPP5E on cilia in RPE1 cells. (A) Immunofluorescence detected the localization of INPP5E in RPE1 cells with siControl (up) or siINPP5E (down) treated and cultured in serum‐free medium for 48 h. (B, C) The fluorescence intensity of INPP5E and Ace‐tubulin in Panel A was assessed using ImageJ. (D) Comparison of the gene sequences after *INPP5E* KO displayed by SnapGene. (E) Synthesized amino acids after *INPP5E* KO displayed by SnapGene. (F) Immunofluorescence detected the localization of INPP5E in RPE1 cells (up) or *INPP5E* KO cells (down) cultured in serum‐free medium for 48 h. (G, H) The fluorescence intensity of INPP5E and Ace‐tubulin in Panel F was assessed using ImageJ. Scale bar, 10 μm.

Immunofluorescence revealed the localization of INPP5E on cilia of RPE1 cells (Figure 2A,B). Knocking down the expression of *INPP5E* in RPE1 cells with siINPP5E resulted in the removal of INPP5E from cilia (Figure 2A,C). Then, we generated *INPP5E* knockout RPE1 cells using CRISPR/Cas9 system. Sequence comparison indicated that the *INPP5E* KO RPE1 cell line lacked a C base (Figure 2D) and translated an incomplete peptide segment (Figure 2E). Consistent with the result of INPP5E deletion by siINPP5E, the localization of INPP5E on cilia also disappeared in *INPP5E* KO RPE1 cells (Figure 2F–H). This implies that the maintenance of PI4P localization on cilia may be dependent on INPP5E.

### 3.3 Deletion of INPP5E Causes PI4P Disappearance from Cilia

**FIGURE 3:**
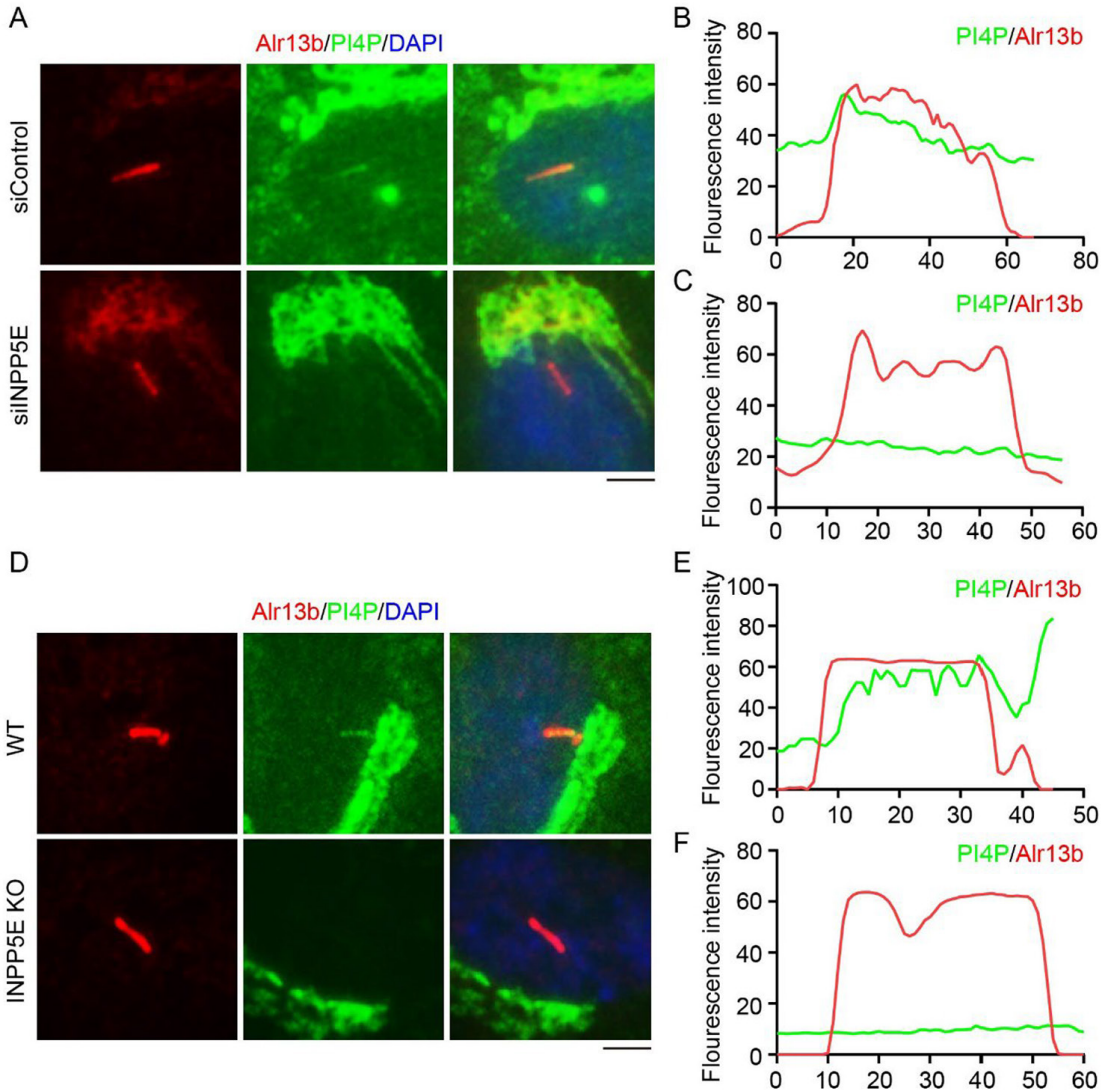
Localization of PI4P on cilia in siRNA‐treated cells or *INPP5E* KO cells. (A) Immunofluorescence detected the localization of PI4P in RPE1 cells treated with siRNA and cultured in serum‐free medium for 48 h. (B, C) The fluorescence intensity of PI4P and Ace‐tubulin in Panel A was assessed using ImageJ. (D) Immunofluorescence detected the localization of PI4P in *INPP5E* KO RPE1 cells cultured in serum‐free medium for 48 h. (E, F) The fluorescence intensity of PI4P and Ace‐tubulin in Panel D was assessed using ImageJ. Scale bar, 10 μm.

To investigate the relationship between INPP5E and the localization of PI4P on cilia, we examined the localization of PI4P on cilia in RPE1 cells treated with siINPP5E. Immunofluorescence results demonstrated that, upon knocking down *INPP5E*, PI4P disappeared from cilia (Figure 3A–C). Consistent with the result of INPP5E deletion by siINPP5E, the localization of PI4P on cilia also vanished in *INPP5E* KO RPE1 cells (Figure 3D–F). These results suggest that INPP5E can change PI4P distribution of ciliary membrane in RPE1 cells.

### 3.4 Deletion of INPP5E Causes the distribution of PI(4,5)P_2_ along Cilia

**FIGURE 4:**
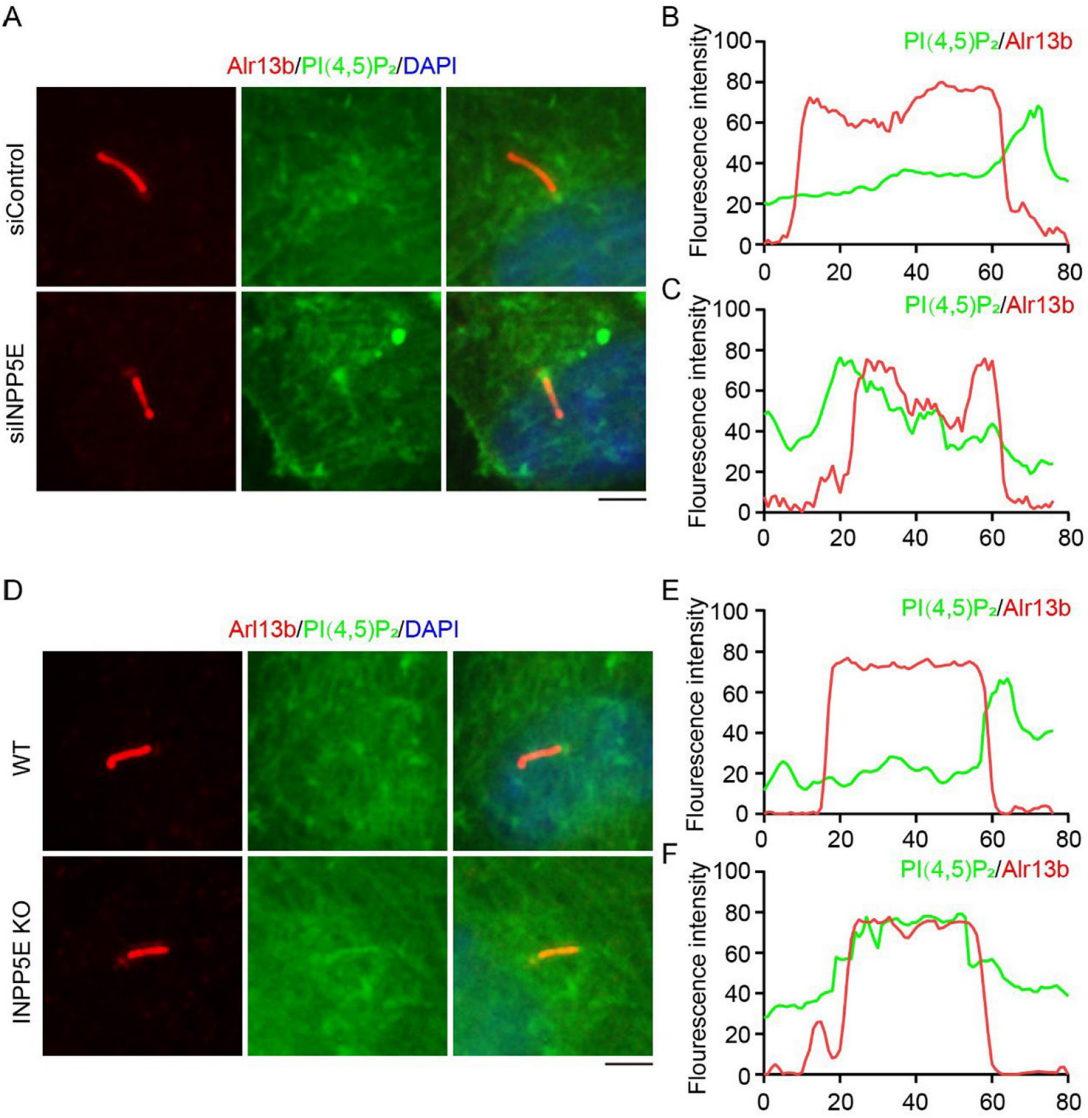
Localization of PI(4,5)P_2_ on cilia in siRNA‐treated cells or *INPP5E* KO cells. (A) Immunofluorescence detected the localization of PI(4,5)P_2_ in RPE1 cells treated with siRNA and cultured in serum‐free medium for 48 h. (B, C) The fluorescence intensity of PI(4,5)P_2_ and Ace‐tubulin in Panel A was assessed using ImageJ. (D) Immunofluorescence detected the localization of PI(4,5)P_2_ in *INPP5E* KO RPE1 cells cultured in serum‐free medium for 48 h. (E, F) The fluorescence intensity of PI(4,5)P_2_ and Ace‐tubulin in Panel D was assessed using ImageJ. Scale bar, 10 μm.

We then aimed to determine whether the localization of PI(4,5)P_2_ changes upon *INPP5E* knockdown. As expected, PI(4,5)P_2_ was found to be localized on the cilia in cells with siINPP5E treated (Figure 4A–C). The same results were observed in *INPP5E* KO cells (Figure 4D–F). This indicates that INPP5E is crucial for the localization of PI4P and PI(4,5)P_2_ on cilia, and may further regulate other cellular processes.

### 3.5 PI(4,5)P_2_ and PI4P Located on Cilia Labeled With SMO

**FIGURE 5:**
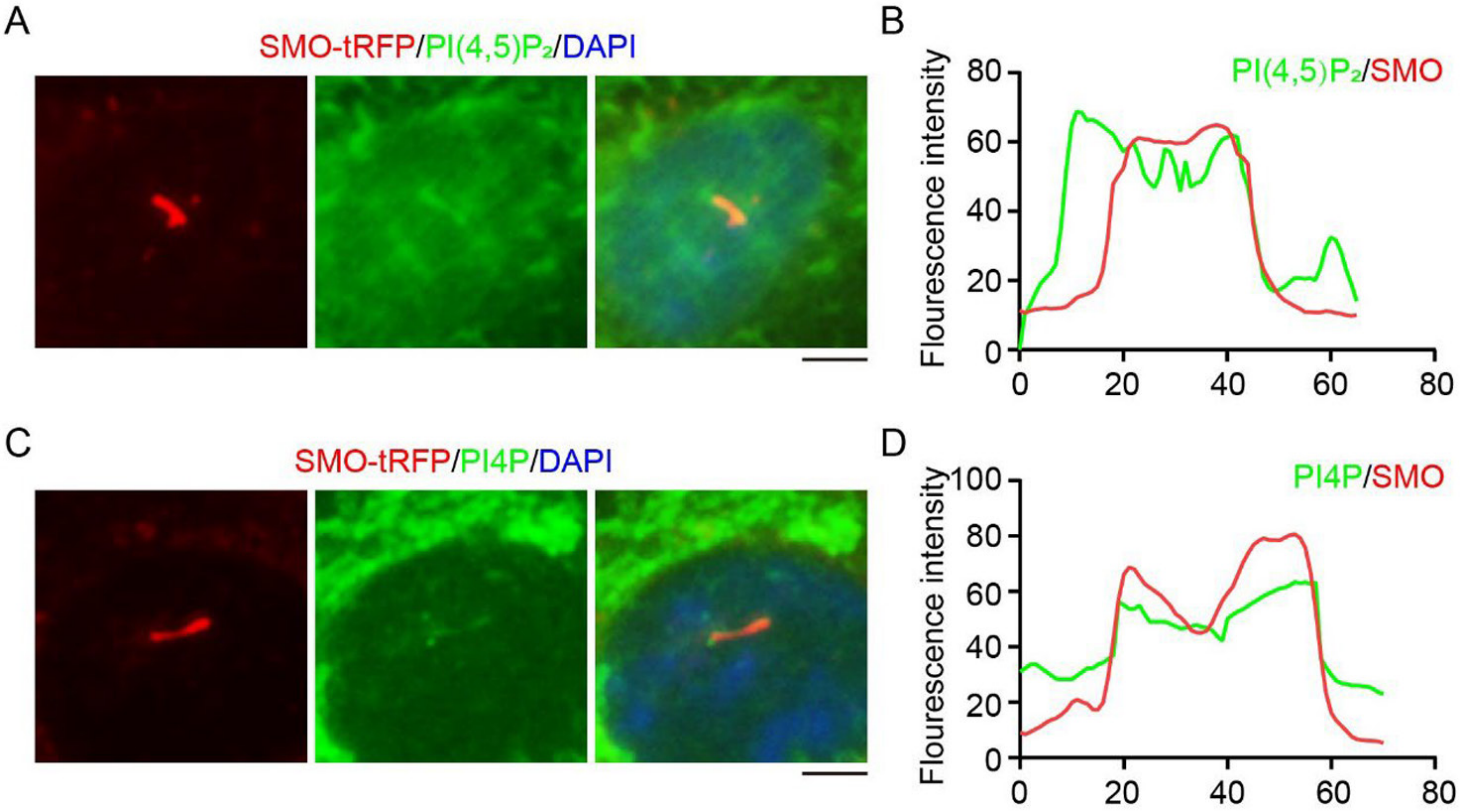
Localization of PI4P and PI(4,5)P_2_ on cilia marked with Smoothened‐tRFP (SMO‐tRFP). (A) Immunofluorescence detected the localization of PI(4,5)P_2_ in SMO‐tRFP RPE1 cells cultured in serum‐free medium for 48 h. (B) The fluorescence intensity of PI(4,5)P_2_ and Ace‐tubulin in Panel A was assessed using ImageJ. (C) Immunofluorescence detected the localization of PI4P in SMO‐tRFP RPE1 cells cultured in serum‐free medium for 48 h. (D) The fluorescence intensity of PI4P and Ace‐tubulin in Panel A was assessed using ImageJ. Scale bar, 10 μm.

Finally, we observed that both PI(4,5)P_2_ and PI4P localize at the ciliary membrane labeled by SMO‐tRFP, which are located on ciliary membrane and can indicate it (Figure 5A–D). The phenomenon of PI(4,5)P_2_ accumulating along the ciliary membrane indicates that this is an intracellular cilia, and the PI component of the ciliary pocket is PI(4,5)P_2_. Further investigation is warranted to ascertain whether alterations in the composition of ciliary pocket phospholipids could impact ciliogenesis.

## 4 Discussion

Phosphoinositide is a derivative of phosphatidylinositol, catalyzed by different kinases and phosphatases [34, 35]. Due to the different temporal and spatial expressions of these kinases and phosphatases, the distribution of different PI in cells is finely regulated [36, 37]. PI and its metabolic pathways play an important role in embryonic development and are an indispensable nutrient crucial for cell growth and survival [38, 39, 40]. INPP5E is a key regulatory enzyme for PI(4,5)P_2_ anabolism in the inositol metabolism pathway and is mainly distributed on primary cilia. The purpose of this study was to mainly explore how INPP5E regulates the major phospholipid composition of cilia. In addition, we demonstrated that knocking down or knocking out INPP5E results in PI(4,5)P_2_ accumulate on primary cilia, while PI4P no longer exists. Phospholipids are crucial for the formation of primary cilia and the stability of mature cilia. Future research should delve into the impact of INPP5E on the distribution of phospholipids in ciliary vesicles, aiming to provide possible mechanisms of INPP5E on ciliogenesis.

Mutations in INPP5E have been identified as causative factors for Bardet–Biedl syndrome (BBS) and Joubert syndrome (JBTS), both characterized by craniofacial abnormalities and skeletal anomalies, and categorized as cilia‐related diseases [41]. This may be closely related to the inositol signaling pathway of primary cilia, acting in the development of neural tube defects [42]. In INPP5E‐deficient embryos, PI(3,4,5)P_3_ is incorrectly distributed to the top surface of cells, and PI(4,5)P_2_ disappears from this region, ultimately leading to defects in ciliogenesis and polycystic kidney disease [43]. Thus, the phosphoinositide distribution regulated by INPP5E is closely associated with ciliopathies. Further investigations are warranted to explore diseases linked to aberrant phosphoinositide metabolism and to uncover potential therapeutic strategies for addressing these conditions.

## Author Contributions

Denghui Zhai, Lamei Li, Cheng Chen, Xue Wang, and Ruming Liu performed the experiments. Denghui Zhai and Lamei Li analyzed the data. Ying Shan conceived and designed the experiments. Denghui Zhai, Lamei Li, and Ying Shan wrote the manuscript.

## Conflicts of Interest

The authors declare no conflicts of interest.

## Data Availability

The data that support the findings of this study are available from the corresponding author upon reasonable request.
